# The Book World of Medicine and Science

**Published:** 1900-10-06

**Authors:** 


### The Book World of Medicine and Science.

Diseases of the Tongue. By Henry T. Butlin, F.R.C.S., D.C.L., Surgeon to St. Bartholomew's Hospital, and Walter G. Spencer, M.S., M.B. (Lond.), F.R.C.S., Surgeon to the Westminster Hospital. Illustrated with eight chromo-lithographs and thirty-six engravings. London: Cassell and Company. 1900. Price 21s.

It was the year 1885 that Mr. Butlin published his well-known work on diseases of the tongue. When, then, after fifteen years' experience and observation he brings out a fresh edition, and does this with collaboration of so well recognised an authority as Mr. Spencer, we naturally expect much from their united labours. And we are not disappointed, for the book before us is not only an admirable study and a careful clinical record of diseases of the tongue, but is a most useful guide to the practitioner in dealing with these often most anxious and difficult cases.

Medical men are probably too ready to regard the state of the tongue as a mere index by which to judge of the condition of the mucous membrane in other portions of the digestive tract. The authors, however, show in how many cases disordered conditions of the tongue are really due to disease, often of an inflammatory nature, in the tongue itself.

It is strongly insisted on, for example, that the fur met with on the tongue consists chiefly of micro-organisms, which indeed form the bulk of the fur; that these are of its essence, and not a mere accidental intrusion; and it is pointed out that the occurrence of changes in the appearance of the tongue is the outcome of local conditions and of variations in the general state of the patient, or in the condition of the blood rather than of any particular state of the digestive organs.

The chapter on chronic superficial glossitis is full of interesting points, including as it does the often anxious ones of smoker's patch and leucoma; and the chromo-lithographs with which it is illustrated are beautiful reproductions of the conditions described. Again, in the chapter on patches, fissures, ulcers, &c., we find admirable clinical descriptions, differentiating conditions with which we are all familiar, and capital pictorial illustrations.

The work done by Mr. Butlin in regard to malignant tumours is so well known that the chapters in which he gives the results of his ripened experience as to the indications for and the utility of operation for cancer of the tongue will be read with much interest, and their teaching will be generally accepted. In a long list of causes which are said to be capable of giving rise to cancer mention is made of "the application of caustics to ulcers and other affections of the tongue," a matter which should be borne in mind by surgeons who may be tempted thus to treat the various ulcerative diseases which come before them. In regard to the question of smoking, the authors say: "We may speak more strongly than we ventured to do some years ago. We believe that smoking is a decided cause of the occurrence of cancer, not so much directly as indirectly; rather by producing, or tending to produce, those conditions on the surface of the tongue which predispose to cancer, than by immediately leading to the development of cancer in such tongues. . . . The common history we receive of much smoking, the great frequency with which cancer of the tongue is preceded by chronic inflamation of the surface of the tongue, which has occurred in smokers and been maintained by smoking, and the much greater liability of males to the disease than females, all lead us to this view."

The authors draw attention to the difficulty which often occurs in differentiating syphilitic lumps and sores, tuberculous ulcers, simple warty tumours, and simple ulcers and fissures from cancer, a difficulty which is much aggravated by the facts that certain of these diseases are sometimes transformed into cancer, and that the transition is by almost imperceptible gradations.

The authors are strongly opposed to the employment of antisyphilitic treatment as a means of diagnosis between syphilis and carcinoma. Such a proceeding is a waste of time at the moment when time is of the greatest importance; and they say that in all cases in which there is ulceration, and the question is raised whether the ulcer is carcinomatous, the microscopic test should be applied, a portion of the wall of the ulcer being removed, cut in sections, and carefully examined. This is to be done under cocaine. "This method of examination should be employed in every doubtful case. It can be used in most instances with success, even when there is no ulceration."

The point is a very important one, and the authors speak very urgently about it, saying that they have seen many cases which showed the harm of letting weeks of valuable time be lost in vain antisyphilitic treatment. "The period at which it should have been removed was allowed to pass, and the operation was undertaken when the prospect of ultimate success was exceedingly small, and when the patient was weakened by the use of large doses of iodide of potassium and, in one case, by mercurial salivation."

Needless to say the operative procedures required are very fully and carefully discussed.

This book is one, the reading of which has given us much pleasure. It is well printed and well bound, the illustrations are strikingly good, and the matter is not only full of interest, but is evidently the outcome of a large experience.

Hernia: its Etiology, Symptoms, and Treatment. By W. McAdam Eccles, M.S. (Lond.), F.R.C.S. (Eng.), Assistant Surgeon West London Hospital, and City of London Truss Society. (London: Baillière, Tindall and Cox. 1900. Price 7s. 6d. net.)

This elaborate and well-illustrated work shows that the days of monographs are not yet over, and also shows the advantages of that form of literature to medical readers. To the man who is only commencing to gather books around him it is well to fill his shelves with works on general surgery, medicine, and the allied sciences. But it is not to such books that he turns when in real difficulty. If he is to get the full help that a library can give, he must be able to consult works which deal far more fully than ordinary text-books ever can do, each with its own subject—books such as this, which, although its author modestly disclaims any attempt at exhaustive treatment, does in fact give just that practical account of hernia, and especially of its modern treatment, which the surgeon wants. After some chapters on the causes of hernia and other general considerations, the clinical conditions of hernia are discussed under the three great divisions of (*a*) reducible hernia; (*b*) hernia which, though irreducible, or inflamed, or obstructed, is still not strangulated; and, finally, (*c*) strangulated hernia. Full and complete directions are given regarding the operative methods which may be required, and an immense amount of detail, profusely illustrated by a large number of capital photographs, is entered into with regard to the application of trusses. To many no doubt this will be found the most interesting part of the book, but the steadily increasing perfection which is being attained in the operation for the radical cure of the ordinary forms of hernia has during recent years very much shifted the standpoint from which this disease has to be regarded, and this book is especially useful in that it enters fully into this the modern operative side of the question. Many important points regarding the relation of hernia to life assurance, accident insurance sick funds, the public services, and residence in tropical climates, are carefully discussed. Altogether the book is likely to be found most useful by its readers, and it certainly does great credit to its author, showing how carefully he has utilised his large opportunities for investigating this disease.

